# Proteome-Wide Lysine Acetylation in Cortical Astrocytes and Alterations That Occur during Infection with Brain Parasite *Toxoplasma gondii*

**DOI:** 10.1371/journal.pone.0117966

**Published:** 2015-03-18

**Authors:** Anne Bouchut, Aarti R. Chawla, Victoria Jeffers, Andy Hudmon, William J. Sullivan

**Affiliations:** 1 Department of Pharmacology & Toxicology, Indiana University School of Medicine, Indianapolis, IN, 46202, United States of America; 2 Department of Microbiology & Immunology, Indiana University School of Medicine, Indianapolis, IN, 46202, United States of America; 3 Department of Biochemistry & Molecular Biology, Indiana University School of Medicine, Indianapolis, IN, 46202, United States of America; Univ. Georgia, UNITED STATES

## Abstract

Lysine acetylation is a reversible post-translational modification (PTM) that has been detected on thousands of proteins in nearly all cellular compartments. The role of this widespread PTM has yet to be fully elucidated, but can impact protein localization, interactions, activity, and stability. Here we present the first proteome-wide survey of lysine acetylation in cortical astrocytes, a subtype of glia that is a component of the blood-brain barrier and a key regulator of neuronal function and plasticity. We identified 529 lysine acetylation sites across 304 proteins found in multiple cellular compartments that largely function in RNA processing/transcription, metabolism, chromatin biology, and translation. Two hundred and seventy-seven of the acetylated lysines we identified on 186 proteins have not been reported previously in any other cell type. We also mapped an acetylome of astrocytes infected with the brain parasite, *Toxoplasma gondii*. It has been shown that infection with *T*. *gondii* modulates host cell gene expression, including several lysine acetyltransferase (KAT) and deacetylase (KDAC) genes, suggesting that the host acetylome may also be altered during infection. In the *T*. *gondii*-infected astrocytes, we identified 34 proteins exhibiting a level of acetylation >2-fold and 24 with a level of acetylation <2-fold relative to uninfected astrocytes. Our study documents the first acetylome map for cortical astrocytes, uncovers novel lysine acetylation sites, and demonstrates that *T*. *gondii* infection produces an altered acetylome.

## Introduction

Astrocytes constitute a major subset of glial cells that carry out a wide variety of critical operations in the mammalian brain. It is well-established that astrocytes play a supportive role for neurons by providing metabolic support, releasing and taking up neurotransmitters, and maintaining extracellular ionic concentrations. Emerging studies suggest that in addition to the long-established structural role astrocytes play in the maintenance of the blood-brain barrier (BBB), astrocytes are key players in neuronal signaling, brain repair, and immune responses [1]. In response to infection, astrocytes release cytokines and chemokines to modulate effector cells. There is also evidence that infection can alter astrocyte function; for example, HIV-infected astrocytes develop abnormal end-feet connections that lead to perturbations in the BBB [2]. Other intracellular microbes capable of infecting astrocytes include *Listeria monocytogenes* and *Toxoplasma gondii* [3–5], but how they may modulate astrocyte function has yet to be defined.

*T*. *gondii* is an obligate, intracellular parasite belonging to the phylum Apicomplexa, which also includes other notorious protozoan pathogens such as *Plasmodium spp*. (malaria) and *Cryptosporidium spp*. (cryptosporidiosis). Up to a third of the world’s population is infected with *T*. *gondii*, which can reactivate as life-threatening disease in immunocompromised individuals. In addition, *T*. *gondii* infection during pregnancy can cause congenital birth defects, including blindness or hydrocephalus [6]. *T*. *gondii* is commonly transmitted to virtually any warm-blooded vertebrate through oocysts expelled by its definitive host (felines) or bradyzoite-containing tissue cysts residing in undercooked meat [7]. The tachyzoite stage of the parasite’s life cycle is characterized by rapid proliferation (doubling time of 6–10 hours) in any nucleated cell and can cause acute disease associated with tissue destruction. In immune competent hosts, the tachyzoites are induced to differentiate into bradyzoites, which are believed to be largely quiescent for the remainder of the host’s life [8]. Attenuation of host immunity can lead to chronic reactivated infection mentioned above.

*T*. *gondii* can traverse the BBB and activate astrocytes as early as 10 days post-infection [9]. While tissue cysts are predominantly seen in neurons during chronic infection, they also occur in microglia and astrocytes during earlier stages of infection [10]. Astrocytes execute several immune functions that are involved in the intracerebral immune response to *T*. *gondii*. With microglia and cerebral microvascular endothelial cells, astrocytes form the IFNγ effector cell population that helps control tachyzoite replication in the brain. IFNγ-activated astrocytes significantly inhibit the growth of tachyzoites in mice via an Immunity-Related GTPase (IRG)-mediated mechanism [11,12] and by expressing pro-inflammatory agents [13–15].

To better understand host-parasite interactions, transcriptional profiling and proteomics analyses have been carried out on *T*. *gondii*-infected versus uninfected cells. *T*. *gondii* infection clearly modulates the host cell, resulting in dramatic changes in host gene expression [16–18] and protein levels [19,20]. Data from Saeij *et al*. reveals that message levels for several lysine acetyltransferases (KATs) and lysine deacetylases (KDACs) are significantly increased (HDAC2 and HAT1) or decreased (SIRT5 and MYST4/MORF) in infected cells [17]. Modulation of KATs and/or KDACs by intracellular pathogens could impact the host cell transcriptome through histone (de)acetylation, thereby modifying the host cell milieu to favor progression of the parasite’s life cycle.

The alteration of host KATs and KDACs is also likely to alter the acetylation status of non-histone proteins as well; we and others have recently found that lysine acetylation is an abundant post-translational modification (PTM) that occurs on thousands of proteins of diverse function throughout multiple cellular compartments [21]. So-called “acetylomes” have now been mapped for several organisms, including bacteria [22,23], plants [24,25], *Saccharomyces cerevisiae* [26] *Drosophila melanogaster* [27], human cells [28,29], and the protozoan parasites *T*. *gondii* and *Plasmodium falciparum* [30–32]. In this study, we present the first proteome-wide analysis of lysine acetylation in cortical astrocytes. We also determined the acetylome of *T*. *gondii*-infected astrocytes, which contained differences relative to the acetylated proteins found in uninfected astrocytes. Our findings add a new layer of complexity to the mechanisms intracellular pathogens may employ to manipulate their host. These studies provide a foundation for follow-up investigations aimed at determining the role of lysine acetylation on individual target proteins and how this impacts astrocytic function as well as *T*. *gondii* pathogenesis.

## Materials and Methods

### Astrocyte cultures

Astrocytes were cultured as previously described [33]. In brief, cortices from postnatal day 1–2 Sprague-Dawley rat pups were enzymatically digested and triturated. Cortical cells were then resuspended in growth media (Dulbecco’s modified Eagle medium (DMEM) containing 5% NuSerum, penicillin 10 units/mL, streptomycin (10μg/ml), L-glutamax (2mM) and B-27) at a density of 2.5 million cells/mL, and resuspended onto 100 μg/ml poly-D-lysine (PDL) coated 10cm dishes. Cells were maintained in humidified incubators at 37°C under 5% CO_2_. Cells were fed every 2–4 days and when the cultures became confluent (7–8 days *in vitro* (DIV)), plates were shaken to remove oligodendrocytes and microglia. After a brief wash, trypsin was used to passage the astrocytes, which were subsequently split onto PDL-coated 10cm dishes (for proteomics or western blotting) or glass coverslips (for immunofluorescence). Similar to previously published findings [33], cultures are negative for MAP-2 (neuronal marker), OX-42 (microglial marker), Olig-2 (oligodendrocyte marker) and GFAP positive (>98%, astrocyte marker).

### *T*. *gondii* culture and astrocyte infection

*T*. *gondii* were maintained in human foreskin fibroblasts (HFF; (ATCC CRL-1634)) using DMEM supplemented with 10% heat-inactivated fetal bovine serum (Invitrogen). RH strain tachyzoites cultured in HFFs were then physically separated from host cells by passage through a 23G syringe needle and purified from host cell debris using a 3.0 μm filter [34]. Following centrifugation, parasites were resuspended in astrocyte growth medium (see above) and used to infect astrocyte monolayers at a multiplicity of infection (MOI) of 10 for 10 h. Several independent preparations had to be pooled in order to obtain 15 mg of protein lysate necessary to generate the astrocyte acetylomes.

### Immunoaffinity enrichment of lysine-acetylated peptides

Harvested astrocytes were washed in PBS and resuspended in urea lysis buffer (9.0 M urea, 20 mM HEPES pH 8.0, 2.5 mM sodium pyrophosphate, 1 mM β-glycerol phosphate, 1 mM sodium orthovanadate) freshly supplemented with 10 mM sodium butyrate, a lysine deacetylase (KDAC) inhibitor. Sonicated lysates were centrifuged for 15 min at 4°C at 20,000 x g. Supernatants were collected and reduced with 4.5 mM DTT for 30 min at 55°C, followed by alkylation with iodoacetimide and dilution with 20 mM HEPES, pH 8.0 to normalize protein concentration across all samples. After digestion with 10 μg/mL trypsin-TPCK in 1.0 mM HCl, peptide lysates were acidified with 1% TFA and peptides were desalted over SEP PAK Classic C18 columns (Waters). Peptides were eluted with 40% acetonitrile in 0.1% TFA, dried under vacuum, and stored at-80°C.

Acetylated peptides were enriched using a pan-specific anti-acetyl-lysine antibody (CST #9895, Cell Signaling Technology) bound to 50 mL packed protein A agarose beads (Roche). Lyophilized peptides were resuspended in MOPS (morpholinepropane sulfonic acid) IAP buffer (50 mM MOPS (pH 7.2), 10 mM KH_2_PO_4_, 50 mM NaCl) and centrifuged for 5 min at 12,000 rpm. Supernatants were mixed with anti-acetyl-lysine beads for 2.5 h at 4°C and then centrifuged for 30 s at 5,400 rpm at 4°C. Beads were washed in MOPS IAP buffer, then in water, prior to elution of the peptides with 0.15% TFA. In preparation for analysis, the peptides were desalted over Empore C_18_ tips (Sigma) and eluted with 60% acetonitrile in 0.1% TFA.

### LC-MS/MS analysis

Liquid chromatography-tandem mass spectrometry (LC-MS/MS) was performed at Cell Signaling Technology (Danvers, MA). Peptides were loaded directly onto a 10 cm x 75 μm PicoFrit capillary column packed with Magic C_18_ AQ reversed-phase resin. The column was developed with a 90-minute linear gradient of acetonitrile in 0.125% formic acid delivered at 280 nL/min. Tandem mass spectra were collected from duplicate samples in a data-dependent manner with an LTQ Orbitrap VELOS mass spectrometer using a top 20 method, a dynamic exclusion repeat count of 1 and a repeat duration of 35 s. MS/MS spectra were evaluated using SEQUEST 3G and the SORCERER 2 platform from Sage-N Research (v4.0, Milpitas CA) [35]. Searches were performed against the most recent update of the NCBI *Rattus norvegicus/Toxoplasma gondii* combined database with mass accuracy of +/-50 ppm for precursor ions and 1 Da for product ions. Results were filtered with mass accuracy of +/− 5 ppm on precursor ions and presence of an acetylated lysine. The mzXML, Dtas, and Out files associated with this study are available upon request. Label-free quantification of individual acetylation sites was performed as previously described, with the fold change for each identified acetylated lysine was calculated by comparing changes in parent peptide ion intensities between uninfected and infected samples [36,37].

### Data analysis and bioinformatics

Acetylated proteins were classified according to gene ontology (GO) annotations by Uniprot (http://www.uniprot.org) [38]. Cellular localization data were also extracted from Uniprot. The secretome analysis was performed using Secretome 2.0 server (http://www.cbs.dtu.dk/services/SecretomeP) [39]. Amino acid sequence motifs were analyzed using WebLogo 3.4 (http://weblogo.threeplusone.com/create.cgi) [40].

### Immunofluorescence assay

Immunofluorescence assays were performed as previously described [41]. Briefly, astrocyte monolayers grown on coverslips were inoculated with RH strain tachyzoites. After removal of culture medium, infected HFFs were fixed in 4% paraformaldehyde for 10 min and then were permeabilized with 0.3% Triton X-100 for 10 min. For visualization of α-tubulin or α-tubulin acetylated at K40, mouse monoclonal anti-α-tubulin antibody (clone DM1A, Sigma T6199) and monoclonal anti-acetylated tubulin antibody (clone 6–11B-1, Sigma T7451) were applied at 1:2,000 followed by goat anti-mouse Alexa Fluor 488 secondary antibody at 1:2,000 (Invitrogen, A-11001). Nuclei were visualized through co-staining with 4′,6-diamidino-2-phenylindole (DAPI).

### Ethics statement

Astrocytes were obtained from postnatal rat pups (DIV1–2) using an approved protocol (10354) from the Institutional Animal Care and Use Committee (IACUC) of the University of Indiana School of Medicine (IUSM). The IUSM is accredited by the International Association for Assessment and Accreditation of Laboratory Animal Care. Animals were anesthetized using volatile anesthetics (halothane/isofluorane) and euthanized by decapitation.

## Results and Discussion

### Proteome-wide analysis of lysine acetylated proteins in cortical astrocytes

As the most abundant type of cell in the mammalian brain, astrocytes carry out a variety of essential functions that include supporting synapse formation, synaptic transmission, and responding to neuronal stress. Astrocytes have been linked to neurodegenerative diseases, such as Alzheimer’s, Parkinson’s, and Huntington’s, as well as amyotrophic lateral sclerosis [42,43]. Efforts to better understand the functions of astrocytes have included recent analyses of the whole-cell proteome and secretome [44–49]. The most recent and comprehensive proteomics study to date was performed using C8-D1A, a type-1 murine astrocyte cell line [50]. Using this cell line, researchers identified 7,183 proteins in the whole-cell astrocyte proteome and 6,067 proteins in the secretome. To date, no one has examined the post-translational modifications (PTMs) that may be decorating the proteome in transformed or dissociated astrocytes.

**Fig 1:**
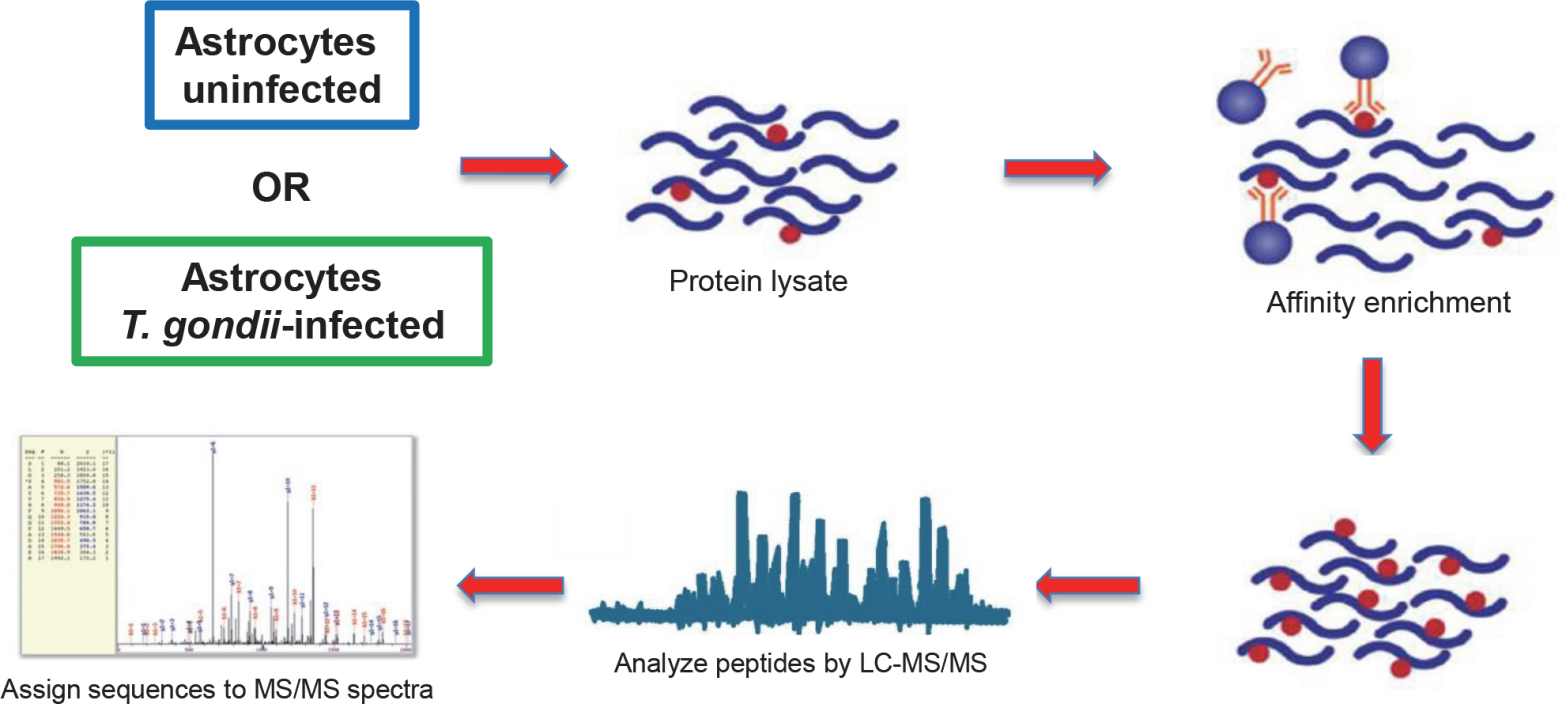
Workflow for acetylome analysis of cortical astrocytes. Protein lysates generated from infected and uninfected cortical astrocytes were subjected to proteolysis to generate a peptide suspension. Immunoaffinity purification with anti-acetyl-lysine antibody enriched for acetylated peptides (acetylation mark is denoted by red dot). Identification of acetylated peptides was achieved with mass spectrometry.

It has now been established for a wide variety of model organisms that thousands of proteins are subject to lysine acetylation throughout cells; however, an acetylome has yet to be determined for any individual brain cell type to date. To address this knowledge gap, and to further examine whether lysine acetylation is altered in response to infection, we performed proteome-wide analyses of lysine acetylated proteins in cortical astrocytes obtained from rat using a strategy outlined in Fig. 1. The significance of charting the astrocyte proteome is underscored by previous studies suggesting that lysine deacetylase (KDAC) inhibitors, such as suberoylanilide hydroxamic acid and valproic acid, show promise in treating some of the aforementioned neurodegenerative diseases in which astrocytes may play a role [51–53]. We first describe the results of the uninfected astrocytes, with the infected astrocyte acetylome discussed in the following section.

Cortical astrocytes were cultured from postnatal day 1–3 Sprague-Dawley rat pups. Generation of whole cell lysates, affinity enrichment of peptides containing acetylated lysine residues, and their identification by mass spectrometry were carried out essentially as described ([30]; see also Materials and Methods). Using this approach, we were able to detect lysine acetyl sites on 516 non-redundant peptides across 304 astrocyte proteins at a false discovery rate (FDR) for peptides of less than 5% (S1 Table). Highlighting the fidelity of our approach and source sample, three marker proteins that distinguish astrocytes from other neuronal cells were detected in our acetylome: fructose-bisphosphate aldolase C (lysine (K) acetylated at amino acid residue 147 (K147)), nuclear factor 1 A-type, NFI-A (K276) and glial fibrillary acidic protein (GFAP) (K258) [50,54,55].

**Fig 2:**
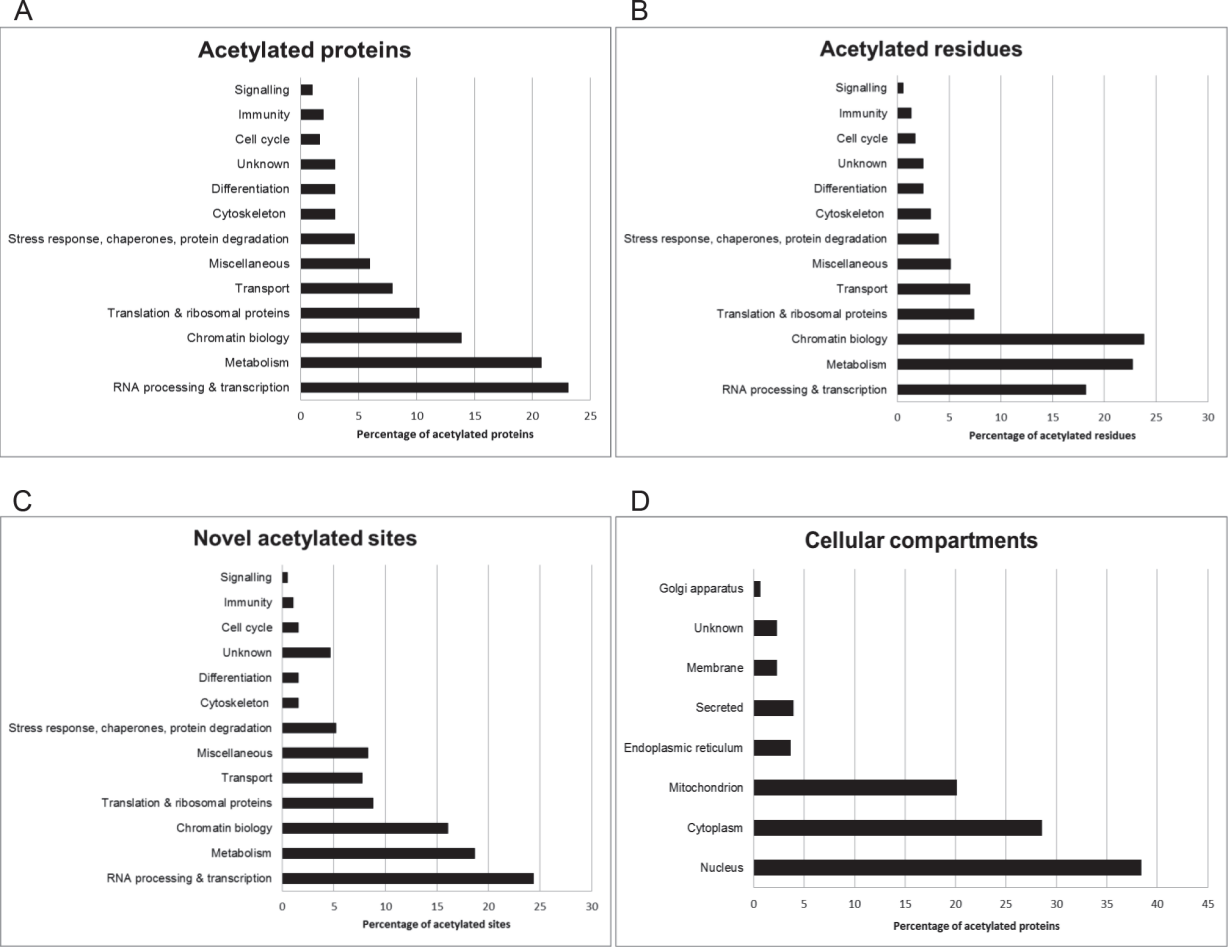
Global features of the cortical astrocyte acetylome. Acetylated proteins (A) and residues (B) were sorted into functional groups based on GO analyses. Lysine acetylation is most prevalent on proteins involved in RNA processing and transcription, metabolism, and chromatin biology. C. The 277 novel acetyl-lysine sites identified in our study are present on proteins that encompass a wide range of cellular functions. D. Proteins detected as lysine-acetylated were grouped based on their respective cellular location.

For a global view of the acetylome, we categorized the acetylated astrocyte proteins into functional groups based on gene ontology (S2 Table). The majority of the acetylated sites and proteins cluster into groups including RNA processing/transcription, metabolism, chromatin biology, and translation (Fig. 2A-B). As expected, histone proteins are heavily acetylated (S3 Table). Acetylation and deacetylation of histones by histone acetyltransferases (HATs) or histone deacetylases (HDACs), respectively, are well-established PTMs that modulate gene expression [56,57].

The first proteomic survey of lysine acetylation was performed in HeLa cells and identified 388 lysine acetylation sites across 195 proteins, which largely clustered into functional categories involving transcription, translation, and metabolism [58], which is comparable to our astrocyte acetylome. A subsequent study performed on human liver identified 1,047 proteins containing lysine acetyl marks, preferentially on those involved in metabolism [29]. A cardiac acetyl-lysine proteome was determined in guinea pigs, revealing that >60% of acetylated proteins are mitochondrial and mainly involved in metabolism, as well as apoptosis and transcription [59]. It is not surprising that different cell types would have variances in the type and abundance of lysine acetylated proteins, but a striking commonality that emerges from these collective acetylomes is the abundance of acetylation on metabolic proteins.

Of the 304 proteins we detected as being acetylated in astrocytes, 186 are novel, having never been previously reported as acetylated in *Rattus novergicus* in Uniprot [38]; thus, our data significantly expands the number of known acetylated lysine sites. The novel lysine acetylation marks we detected were found primarily on proteins associated with RNA processing and transcription, metabolism, chromatin biology, and translation (Fig. 2C).

We examined the presence of acetylated proteins in different subcellular compartments. Our data reveals that proteins subject to lysine acetylation in astrocytes are predominantly found in the nucleus (38.5%), followed by the cytoplasm (28%) and mitochondria (20%). Eleven of the acetylated proteins (3.6% of the acetylome) are secreted from astrocytes (Fig. 2D).

One of the secreted proteins which we identified as acetylated is a complement protein involved in innate immunity: C3, which is acetylated on K215 within the N-terminal beta chain region. Complement C3 has not previously been reported to be acetylated, so it is unclear what the function of this acetylation site may be. It is possible that acetylation of complement C3 could alter folding of the protein and affect secretion and/or cleavage of C3 by C3 convertase, thus regulating the downstream complement cascade. This is particularly intriguing considering our finding that this acetyl mark is increased 3.3-fold upon *T*. *gondii* infection (see below), lending support to the idea that acetylation of K215 on C3 may modulate complement function. Treatment with the lysine deacetylase (KDAC) inhibitors sodium butyrate and Trichostatin A has been shown to enhance complement C3 expression and secretion, through increased histone acetylation [60], but the direct action of KDACs on complement C3 itself was not considered.

Transmembrane glycoprotein NMB precursor (Gpnmb) was another secreted protein on which a novel acetylation site was identified, K170. Also known as osteoclavin in rats, this highly glycosylated protein is anchored in the plasma membrane and has been shown to regulate cellular differentiation, specifically the development of osteoblasts in bone [61]. Gpnmb was also found to be upregulated in both neurons and astrocytes after brain ischaemic injury in rats and overexpression of Gpnmb was protective against ischaemic injury [62], indicating a role for Gpnmb in neuroprotection. Acetylation of Gpnmb may be involved in the regulation of these neural repair processes, through controlling Gpnmb secretion or glycosylation.

**Fig 3:**
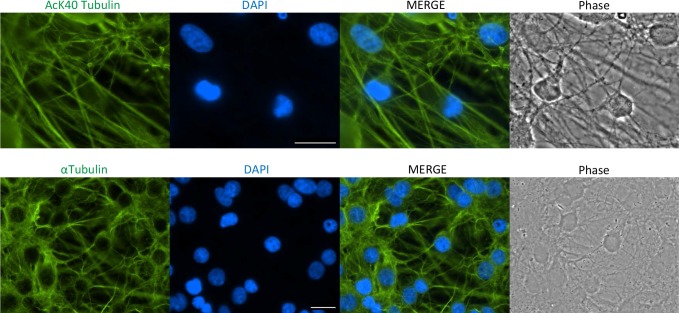
Localization of acetylated tubulin in astrocytes. The subcellular localization of α-tubulin acetylated at K40 (upper panels) and total α-tubulin (lower panels) through immunofluorescence of isolated cortical astrocytes (green). DAPI staining to highlight nuclei is indicated in blue. Scale bar = 20 μm.

As mentioned above, histone proteins are known to be heavily acetylated and this is no exception in astrocytes (S3 Table). Unexpectedly, we found novel histone acetylation sites on some astrocyte histones. For example, linker histone H1C is acetylated at K17, K75, and K84, in addition to the previously detected K46, K90, and K106 (http://www.phosphosite.org/proteinAction.do?id=3849&showAllSites=true) [63]. The most abundant acetylated non-histone protein in cultured cortical rat astrocytes is α-tubulin, which we detected as acetylated at K40. K40 acetylation is widespread among eukaryotes and its role in the cell remains a topic of intense investigation. Acetylated tubulin is a feature of long-lived, stable microtubules [64] and may interplay with additional PTMs to form a “tubulin code” that provides microtubules flexibility to execute a wide variety of cellular functions [65]. We performed immunofluorescent analysis using an antibody specific against the acetylated K40 residue of tubulin, which confirms that acetylated microtubules are abundant in cortical astrocytes and localized uniformly throughout the cell (Fig. 3).

A KEGG pathway analysis using DAVID (Database for Annotation, Visualization and Integrated Discovery; http://david.abcc.ncifcrf.gov/home.jsp) [66,67] of the astrocyte acetylome clustered 123 of the 304 acetylated proteins as proteins linked to neurological disorders, such as Huntington’s disease (18 proteins), Parkinson’s disease (15 proteins), and Alzheimer’s disease (12 proteins) (Table 1 and S4 Table). Acetylated proteins are also associated with canonical metabolic pathways that have been shown to be specifically enriched in astrocytes compared to neurons and oligodendrocytes [68], including the citrate cycle, fatty acid metabolism, and valine, leucine, and isoleucine degradation (S4 Table).

**Table 1:** Proteins involved in neurological disorders that are acetylated in murine astrocytes.

Neurological disorder	Accession number	Protein name
**Huntington’s Disease**	Q9Z2L0	voltage-dependent anion-selective channel protein 1
	P10719	ATP synthase subunit beta, mitochondrial precursor
	P35434	ATP synthase subunit delta, mitochondrial precursor
	B2RYS2	cytochrome b-c1 complex subunit 7
	Q4QQW4	histone deacetylase 1
	P81155	voltage-dependent anion-selective channel protein 2
	P19511	ATP synthase subunit b, mitochondrial precursor
	P29418	ATP synthase subunit epsilon, mitochondrial
	P15999	ATP synthase subunit alpha, mitochondrial precursor
	P31399	ATP synthase subunit d, mitochondrial
	Q09073	ADP/ATP translocase 2
	Q920L2	succinate dehydrogenase [ubiquinone] flavoprotein subunit, mitochondrial precursor
	Q6JHU9	CREB-binding protein
	Q05962	ADP/ATP translocase 1
	Q5M9I5	cytochrome b-c1 complex subunit 6, mitochondrial
	P07895	superoxide dismutase [Mn], mitochondrial precursor
	Q9R1Z0	voltage-dependent anion-selective channel protein 3
	Q06647	ATP synthase subunit O, mitochondrial precursor
**Parkinson’s Disease**	Q9Z2L0	voltage-dependent anion-selective channel protein 1
	P10719	ATP synthase subunit beta, mitochondrial precursor
	P35434	ATP synthase subunit delta, mitochondrial precursor
	B2RYS2	cytochrome b-c1 complex subunit 7
	P81155	voltage-dependent anion-selective channel protein 2
	P19511	ATP synthase subunit b, mitochondrial precursor
	P29418	ATP synthase subunit epsilon, mitochondrial
	P15999	ATP synthase subunit alpha, mitochondrial precursor
	P31399	ATP synthase subunit d, mitochondrial
	Q09073	ADP/ATP translocase 2
	Q920L2	succinate dehydrogenase [ubiquinone] flavoprotein subunit, mitochondrial precursor
	Q05962	ADP/ATP translocase 1
	Q5M9I5	cytochrome b-c1 complex subunit 6, mitochondrial
	Q9R1Z0	voltage-dependent anion-selective channel protein 3
	Q06647	ATP synthase subunit O, mitochondrial precursor
**Alzheimer’s Disease**	P19511	ATP synthase subunit b, mitochondrial precursor
	P15999	ATP synthase subunit alpha, mitochondrial precursor
	P29418	ATP synthase subunit epsilon, mitochondrial
	P31399	ATP synthase subunit d, mitochondrial
	P10719	ATP synthase subunit beta, mitochondrial precursor
	P35434	ATP synthase subunit delta, mitochondrial precursor
	P62161	calmodulin
	B2RYS2	cytochrome b-c1 complex subunit 7
	Q920L2	succinate dehydrogenase [ubiquinone] flavoprotein subunit, mitochondrial precursor
	P04797	glyceraldehyde-3-phosphate dehydrogenase
	Q5M9I5	cytochrome b-c1 complex subunit 6, mitochondrial
	Q06647	ATP synthase subunit O, mitochondrial precursor

**Fig 4:**
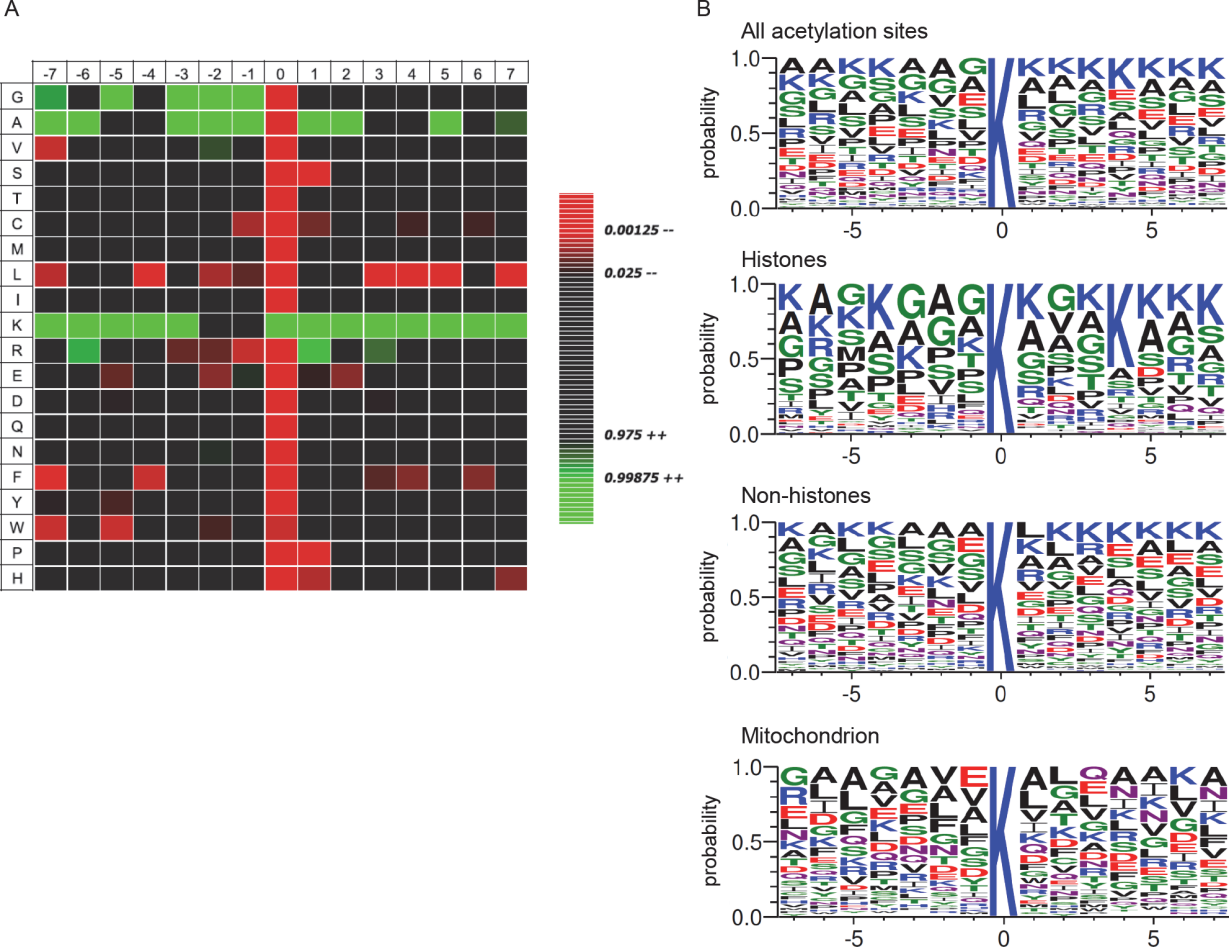
Analysis of acetylated lysine sites. A. Heat map of amino acid composition of acetylation sites in *Rattus norvegicus* astrocytes, displaying amino acids that are significantly enriched (green) or absent (red) relative to the general amino acid composition of the *Rattus norvegicus* proteome. B. Sequence motifs of astrocyte acetylation sites +/-7 amino acids from the targeted lysine residue. Motifs were compiled using all acetylated peptides or only those found in histones, non-histone proteins, or mitochondrial proteins.

We also assessed if the amino acids flanking the targeted acetyl-lysine exhibits bias towards a certain motif and if there is significant enrichment or absence of specific amino acids with respect to the general amino acid composition of the entire *Rattus norvegicus* proteome. For these analyses, we generated WebLogo sequence motifs [40] and IceLogo heat maps [69]. As previously reported for multiple cell types, we found that lysine acetylation of astrocyte proteins also generally occurs in lysine-rich regions, with a significant enrichment for glycine and alanine at positions-1, -2, and-3 and positions +1, +2, and +5 for alanine only, as shown in Fig. 4A [27,28]. The heat map also shows an absence of serine, proline, and histidine at position +1, and a general lack of leucine in the vicinity of the acetylation site. However, the preponderance of histone proteins in acetylome datasets likely biases the global motif analysis; searching for only the acetylated proteins in mitochondria reveals a striking deviance from other acetylation sites (Fig. 4B). In the acetylated proteins in astrocyte mitochondria, there is a modest enrichment for glutamate (E) at the-1 position, which has been observed before in multiple studies [70]. Analysis of the histone proteins alone revealed a high conservation of the GK motif, with additional lysines at the + and—4 positions, consistent with acetylated histones in other species and cell types [28,58]. Our data lends support to the idea that different motifs are targeted by distinct KATs residing in that particular cellular compartment [70].

### Differences in lysine acetylation in astrocytes infected with *T*. *gondii*

The results above establish that many other proteins in addition to histones are subject to lysine acetylated in astrocytes. We also addressed how the acetylome in cortical astrocytes is impacted by *T*. *gondii* infection. In parallel studies, we performed an acetylome on astrocytes that were infected with RH strain *T*. *gondii* tachyzoites for 10 hours, at which point vacuoles contain 2–4 parasites. Compared to the uninfected astrocytes, 58 proteins were differentially acetylated when infected with *T*. *gondii*. Table 2 lists the 34 proteins that exhibit at least a 2-fold increase in lysine acetylation; 9 of these proteins contain 10 novel acetylated lysines that have yet to be reported.

**Table 2:** Proteins showing ≥2-fold increase in lysine acetylation in *T. gondii*-infected astrocytes.

Functional Group	Accession number	Protein name	Normalized Fold-change
			*T. gondii*:control
**Chromatin Biology**	P62804	histone H4	2.2
	XP_001054684	histone H4 replacement-like	2.4
	P84245	histone H3.3B-like	4.4
	P0C0S7	histone H2A.Z	11.5
	Q6LED0	histone H3.3	665.7
**Differentiation**	F1LRS2	dedicator of cytokinesis 7	3.3
**Immunity**	P01026	complement C3	3.3
**Metabolism**	P97519	hydroxymethylglutaryl-CoA lyase, mitochondrial precursor	2.1
	P04636	malate dehydrogenase, mitochondrial precursor	2.1
	P17764	acetyl-CoA acetyltransferase, mitochondrial precursor	2.1
	P12785	fatty acid synthase	2.3
	P29411	GTP:AMP phosphotransferase, mitochondrial	2.4
	Q811I0	ATP synthase mitochondrial F1 complex assembly factor 1	3.4
	P15999	ATP synthase subunit alpha, mitochondrial precursor	4.2
**Miscellaneous**	P07895	superoxide dismutase [Mn], mitochondrial precursor	2.1
	P48679	prelamin-A/C isoform C2	2.7
	P07895	superoxide dismutase [Mn], mitochondrial precursor	5.4
	Q6P7C7	transmembrane glycoprotein NMB precursor	136
**RNA processing & transcription**	B0BNB4	mediator of RNA polymerase II transcription subunit 6	2.2
	Q66H19	serum response factor-binding protein 1	2.2
	Q6IMY8	heterogeneous nuclear ribonucleoprotein U	3
	P09416	myc proto-oncogene protein (c-Myc)	3
	P0C1G9	transcription factor SOX-11	3.5
	Q5TKR9	histone acetyltransferase MYST3	5.2
	Q6JHU9	CREB-binding protein (CBP)	6.6
	Q99MK2	histone acetyltransferase KAT5/TIP60	6.9
**Stress response, chaperones, protein degradation**	P26772	10 kDa heat shock protein, mitochondrial	2
	P14659	heat shock-related 70 kDa protein 2	2.3
**Translation & ribosomal proteins**	Q3T1J1	eukaryotic translation initiation factor 5A-1	2.8
	P18445	60S ribosomal protein L27a	4.5
**Transport**	Q75Q39	mitochondrial import receptor subunit TOM70	2.1
	P61972	nuclear transport factor 2	2.9
	P02770	serum albumin precursor	6.3
**Unknown**	Q5RKH0	putative oxidoreductase GLYR1	2

Notably, several of the acetylated proteins in the infected astrocytes are transcription factors and KATs themselves, which may be involved with the profound changes in gene expression programs previously observed in other *T*. *gondii*-infected cells. Consistent with the up-regulation of host cell genes upon *T*. *gondii* invasion, hyperacetylation of canonical histones is observed, particularly on H3.3, which shows a remarkable 600-fold increase in lysine acetylation (Table 2). Such a large effect on histone acetylation suggests that modulations in host cell gene expression are largely driven at the epigenetic level.

The greatest increase in acetylation in response to *T*. *gondii* infection on a non-histone protein occurs on K170 of GPNMB/osteoactivin, a type I transmembrane glycoprotein nonmetastatic melanoma B (136-fold increase, Table 2). GPNMB plays multiple roles, including bone regeneration, tumor growth regulation, and T-cell inactivation [71–73]. In astrocytes, GPNMB is secreted as a neuroprotective factor [62,74]. As GPNMB is also important for tissue repair [75], this protein may help manage the damage done by the parasite. The role of acetylation on GPNMB is unknown, and there has been no previous link between this protein and *T*. *gondii* infection.

Increased acetylation in select proteins during infection may be due in part to acetylation of KATs like CBP and TIP60 themselves, which display a 6.6- and 6.9-fold increase, respectively (Table 2). K327 acetylation, for example, has been linked to maximizing TIP60 enzyme activity [76]. TIP60 has also been shown to interact with and acetylate c-Myc, dramatically enhancing the stability of c-Myc protein [77]. It is tempting to speculate that *T*. *gondii* infection modulates these KAT enzymes, which then leads to hyperacetylation of their corresponding substrates.

Two dozen astrocyte proteins exhibited 2-fold less acetylated lysine levels in the acetylome from *T*. *gondii*-infected astrocytes (Table 3), and 28 of the acetylated lysines across these 24 proteins have never been reported in Uniprot. Nearly 30% of the decreased acetylation appears on variant histones, suggesting that *T*. *gondii* infection of astrocytes is a large driver of changes in host gene expression. Previous studies have shown that *T*. *gondii* infection results in decreased acetylation of core histones H3 and H4 located at the promoters of IFNγ-regulated genes [78]. However, no studies have examined the roles of histone variants in host cells infected with *T*. *gondii*. Our results suggest that the rewiring of host cell gene expression in response to infection is highly sophisticated and may involve an opposing interplay of acetylation on canonical versus variant histones.

**Table 3:** Proteins showing ≤2-fold decrease in lysine acetylation in *T. gondii*-infected astrocytes.

Functional group	Accession number	Protein name	Normalized Fold-change
			*T. gondii*:control
**Cell cycle**	B2RYQ5	enhancer of rudimentary homolog	-2.3
**Chromatin Biology**	D3ZWM5	histone cluster 1, H2bd-like	-2.6
	Q9Z2Q9	histone H2B	-2.6
	Q63945	protein SET	-2.7
	D3ZLY9	histone cluster 1, H2bp	-3.1
	G3V8B3	histone cluster 1, H2bm	-3.5
	P62804	histone cluster 1, H4m	-3.7
	M0RCB8	histone H3.3B-like	-6
	D3ZNZ9	histone cluster 3, H2ba	-10.1
**Cytoskeleton**	P68370	tubulin alpha-1A chain	-2.3
**Differentiation**	P62161	calmodulin	-2.4
**Metabolism**	Q9EQS0	transaldolase	-2.3
	P16617	phosphoglycerate kinase 1	-3
	P56574	isocitrate dehydrogenase [NADP], mitochondrial precursor	-10.2
**Miscellaneous**	P10111	peptidyl-prolyl cis-trans isomerase A	-2.2
**RNA processing & transcription**	A7VJC4	heterogeneous nuclear ribonucleoproteins A2/B1	-2
	B0BNB4	chromatin modification-related protein MEAF6	-2.1
	Q4V898	heterogeneous nuclear ribonucleoprotein G	-2.3
	A1L130	inhibitor of growth protein 4	-2.4
	D4A411	peregrin	-11.3
**Stress response, chaperones, protein degradation**	Q91XW0	heat shock protein HSP90-alpha	-19.2
**Translation & ribosomal proteins**	Q68FR6	elongation factor 1-gamma	-2.2
**Transport**	P02693	fatty acid-binding protein, intestinal	-2.1
	Q05962	ADP/ATP translocase 1	-7

The most dramatic reduction of lysine acetylation was observed for K408 of the heat shock protein Hsp90α, exhibiting a 19.2-fold decrease in the acetylome of infected astrocytes (Table 3). Hsp90α is an essential molecular chaperone that activates multiple client proteins typically in response to cellular stress [79]. Activation of Hsp90α and interactions with its client proteins and co-chaperones are mediated by several PTMs including lysine acetylation [80]. The acetylation of K408 has not been reported to date, but it was detected as ubiquitylated in mice and humans [81,82], making it possible that acetylation here prevents Hsp90α degradation. The dramatic reduction in deacetylation of K408 suggests that Hsp90α may be suppressed in response to *T*. *gondii* infection.

Another protein with greatly reduced acetylation (<10-fold) in the infected astrocyte acetylome is isocitrate dehydrogenase (IDH2), at K180. As a supplier of NADPH, IDH2 is a critical component of the mitochondrial antioxidant pathway [83], and is necessary for the regeneration of reduced glutathione (GSH), the major antioxidant responsible for preventing ROS damage [84]. IDH2 acetylation at K143 results in a 44-fold loss in activity; deacetylation by SIRT3 restores IDH2 activity and protects cells from oxidative stress [85]. It is possible that *T*. *gondii* mediates a deacetylation of IDH2 to protect its host cell from oxidative or other stresses associated with managing infection, but further studies are required to determine the role this lysine plays in IDH2 function in both infected and uninfected astrocytes.

## Conclusions

Recent studies have implicated important roles for lysine acetylation in other cell types during viral infection that involve interference with host cell KATs [86], hijacking of host cell acetylome machinery or acetylation of viral proteins [87–91], and other changes in lysine acetylation during infection or in response to viral pathogens [92,93]. Intracellular bacteria also effect host cell lysine acetylation. For example, *Salmonella enterica* appear to exploit the host TIP60 KAT activity to promote efficient replication inside host cells [94]. Additionally, *Salmonella typhimurium* infection increases p53 acetylation in intestinal epithelial cells [95]. Our study lends support to the idea that intracellular pathogens modulate host cell proteomes and PTMs such as lysine acetylation. Previously, it has also been shown that host cell proteins can be differentially phosphorylated in response to *T*. *gondii* infection [19].

It is important to note that some of the changes in acetylation detected in the infected astrocytes could potentially be reflective of changes in the abundance of that protein induced by the infection. Future studies are required to elucidate the biological consequences of the changes we detected in the astrocyte acetylome upon infection with *T*. *gondii*, as alterations in lysine acetylation status could affect protein localization, function, stability, or interactions [96].

*T*. *gondii* secretes numerous proteins that alter host cell protein phosphorylation [97–99]. *T*. *gondii* possesses several KATs and KDACs, but there is no evidence that these are secreted. A more likely mechanism is that *T*. *gondii* effector proteins modulate host KATs and KDACs activities that lead to altered acetylation patterns. Our datasets provide a wealth of new information, including 186 novel lysine acetylation sites to add to the cellular inventory of PTMs. These data serve as valuable resources to generate hypotheses about astrocyte physiology and to interrogate how the changes in host cell lysine acetylation may contribute to the effectiveness of *T*. *gondii* infection.

## Supporting Information

S1 Table Astrocyte acetylome. (XLSX)

S2 Table Classification of acetylated astrocyte proteins into functional groups. (XLSX)

S3 Table Acetylated histone proteins in astrocytes. (XLSX)

S4 Table KEGG pathway enrichment of acetylated astrocyte proteins. (XLSX)

## References

[1] Fields RD, Stevens-Graham B (2002) New insights into neuron-glia communication. Science 298: 556–562. 12386325 10.1126/science.298.5593.556 PMC1226318

[2] Eugenin EA, Clements JE, Zink MC, Berman JW (2011) Human immunodeficiency virus infection of human astrocytes disrupts blood-brain barrier integrity by a gap junction-dependent mechanism. J Neurosci 31: 9456–9465. 10.1523/JNEUROSCI.1460-11.2011 21715610 PMC3132881

[3] Dramsi S, Levi S, Triller A, Cossart P (1998) Entry of Listeria monocytogenes into neurons occurs by cell-to-cell spread: an in vitro study. Infect Immun 66: 4461–4468. 9712801 10.1128/iai.66.9.4461-4468.1998 PMC108539

[4] Sims TA, Hay J, Talbot IC (1989) An electron microscope and immunohistochemical study of the intracellular location of Toxoplasma tissue cysts within the brains of mice with congenital toxoplasmosis. Br J Exp Pathol 70: 317–325. 2504268 PMC2040570

[5] Halonen SK, Lyman WD, Chiu FC (1996) Growth and development of Toxoplasma gondii in human neurons and astrocytes. J Neuropathol Exp Neurol 55: 1150–1156. 8939198 10.1097/00005072-199611000-00006

[6] Montoya JG, Liesenfeld O (2004) Toxoplasmosis . Lancet 363: 1965–1976. 15194258 10.1016/S0140-6736(04)16412-X

[7] Hill DE, Chirukandoth S, Dubey JP (2005) Biology and epidemiology of Toxoplasma gondii in man and animals. Anim Health Res Rev 6: 41–61. 16164008 10.1079/ahr2005100

[8] Sullivan WJ Jr, Jeffers V (2012) Mechanisms of Toxoplasma gondii persistence and latency. FEMS microbiology reviews 36: 725–733. 10.1111/j.1574-6976.2011.00305.x PMC3319474 22091606

[9] Hunter CA, Roberts CW, Alexander J (1992) Kinetics of cytokine mRNA production in the brains of mice with progressive toxoplasmic encephalitis. Eur J Immunol 22: 2317–2322. 1516621 10.1002/eji.1830220921

[10] Ferguson DJ, Hutchison WM (1987) An ultrastructural study of the early development and tissue cyst formation of Toxoplasma gondii in the brains of mice. Parasitol Res 73: 483–491. 3422976 10.1007/BF00535321

[11] Halonen SK, Taylor GA, Weiss LM (2001) Gamma interferon-induced inhibition of Toxoplasma gondii in astrocytes is mediated by IGTP. Infect Immun 69: 5573–5576. 11500431 10.1128/IAI.69.9.5573-5576.2001 PMC98671

[12] Scheidegger A, Vonlaufen N, Naguleswaran A, Gianinazzi C, Muller N, et al (2005) Differential effects of interferon-gamma and tumor necrosis factor-alpha on Toxoplasma gondii proliferation in organotypic rat brain slice cultures. J Parasitol 91: 307–315. 15986605 10.1645/GE-379R

[13] Wilson EH, Hunter CA (2004) The role of astrocytes in the immunopathogenesis of toxoplasmic encephalitis. Int J Parasitol 34: 543–548. 15064118 10.1016/j.ijpara.2003.12.010

[14] Drogemuller K, Helmuth U, Brunn A, Sakowicz-Burkiewicz M, Gutmann DH, et al (2008) Astrocyte gp130 expression is critical for the control of Toxoplasma encephalitis. J Immunol 181: 2683–2693. 18684959 10.4049/jimmunol.181.4.2683

[15] Strack A, Asensio VC, Campbell IL, Schluter D, Deckert M (2002) Chemokines are differentially expressed by astrocytes, microglia and inflammatory leukocytes in Toxoplasma encephalitis and critically regulated by interferon-gamma. Acta Neuropathol 103: 458–468. 11935261 10.1007/s00401-001-0491-7

[16] Blader IJ, Manger ID, Boothroyd JC (2001) Microarray analysis reveals previously unknown changes in Toxoplasma gondii-infected human cells. J Biol Chem 276: 24223–24231. 11294868 10.1074/jbc.M100951200

[17] Saeij JP, Coller S, Boyle JP, Jerome ME, White MW, et al (2007) Toxoplasma co-opts host gene expression by injection of a polymorphic kinase homologue. Nature 445: 324–327. 17183270 10.1038/nature05395 PMC2637441

[18] Melo MB, Nguyen QP, Cordeiro C, Hassan MA, Yang N, et al (2013) Transcriptional analysis of murine macrophages infected with different Toxoplasma strains identifies novel regulation of host signaling pathways. PLoS Pathog 9: e1003779 10.1371/journal.ppat.1003779 24367253 PMC3868521

[19] Nelson MM, Jones AR, Carmen JC, Sinai AP, Burchmore R, et al (2008) Modulation of the host cell proteome by the intracellular apicomplexan parasite Toxoplasma gondii. Infect Immun 76: 828–844. 17967855 10.1128/IAI.01115-07 PMC2223483

[20] Zhou DH, Yuan ZG, Zhao FR, Li HL, Zhou Y, et al (2011) Modulation of mouse macrophage proteome induced by Toxoplasma gondii tachyzoites in vivo. Parasitol Res 109: 1637–1646. 10.1007/s00436-011-2435-z 21584632

[21] Rao RS, Thelen JJ, Miernyk JA (2014) Is Lys-N(varepsilon)-acetylation the next big thing in post-translational modifications? Trends Plant Sci 19: 550–553. 10.1016/j.tplants.2014.05.001 24866592

[22] Pan J, Ye Z, Cheng Z, Peng X, Wen L, et al (2014) Systematic analysis of the lysine acetylome in Vibrio parahemolyticus. J Proteome Res 13: 3294–3302. 10.1021/pr500133t 24874924

[23] Hu LI, Lima BP, Wolfe AJ (2010) Bacterial protein acetylation: the dawning of a new age. Molecular microbiology 77: 15–21. 10.1111/j.1365-2958.2010.07204.x 20487279 PMC2907427

[24] Finkemeier I, Laxa M, Miguet L, Howden AJ, Sweetlove LJ (2011) Proteins of diverse function and subcellular location are lysine acetylated in Arabidopsis. Plant physiology 155: 1779–1790. 10.1104/pp.110.171595 21311031 PMC3091095

[25] Wu X, Oh MH, Schwarz EM, Larue CT, Sivaguru M, et al (2011) Lysine acetylation is a widespread protein modification for diverse proteins in Arabidopsis. Plant physiology 155: 1769–1778. 10.1104/pp.110.165852 21311030 PMC3091122

[26] Weinert BT, Iesmantavicius V, Moustafa T, Scholz C, Wagner SA, et al (2014) Acetylation dynamics and stoichiometry in Saccharomyces cerevisiae. Mol Syst Biol 10: 716 10.1002/msb.134766 24489116 PMC4023402

[27] Weinert BT, Wagner SA, Horn H, Henriksen P, Liu WR, et al (2011) Proteome-wide mapping of the Drosophila acetylome demonstrates a high degree of conservation of lysine acetylation. Science signaling 4: ra48 10.1126/scisignal.2001902 21791702

[28] Choudhary C, Kumar C, Gnad F, Nielsen ML, Rehman M, et al (2009) Lysine acetylation targets protein complexes and co-regulates major cellular functions. Science 325: 834–840. 10.1126/science.1175371 19608861

[29] Zhao S, Xu W, Jiang W, Yu W, Lin Y, et al (2010) Regulation of cellular metabolism by protein lysine acetylation. Science 327: 1000–1004. 10.1126/science.1179689 20167786 PMC3232675

[30] Jeffers V, Sullivan WJ Jr (2012) Lysine Acetylation Is Widespread on Proteins of Diverse Function and Localization in the Protozoan Parasite Toxoplasma gondii. Eukaryot Cell 11: 735–742. 10.1128/EC.00088-12 22544907 PMC3370464

[31] Xue B, Jeffers V, Sullivan WJ, Uversky VN (2013) Protein intrinsic disorder in the acetylome of intracellular and extracellular Toxoplasma gondii. Mol Biosyst 9: 645–657. 10.1039/c3mb25517d 23403842 PMC3594623

[32] Miao J, Lawrence M, Jeffers V, Zhao F, Parker D, et al (2013) Extensive lysine acetylation occurs in evolutionarily conserved metabolic pathways and parasite-specific functions during Plasmodium falciparum intraerythrocytic development. Mol Microbiol 89: 660–675. 10.1111/mmi.12303 23796209 PMC3757501

[33] Ashpole NM, Chawla AR, Martin MP, Brustovetsky T, Brustovetsky N, et al (2013) Loss of calcium/calmodulin-dependent protein kinase II activity in cortical astrocytes decreases glutamate uptake and induces neurotoxic release of ATP. J Biol Chem 288: 14599–14611. 10.1074/jbc.M113.466235 23543737 PMC3656312

[34] Roos DS, Donald RG, Morrissette NS, Moulton AL (1994) Molecular tools for genetic dissection of the protozoan parasite Toxoplasma gondii. Methods Cell Biol 45: 27–63. 7707991 10.1016/s0091-679x(08)61845-2

[35] Lundgren DH, Martinez H, Wright ME, Han DK (2009) Protein identification using Sorcerer 2 and SEQUEST. Current protocols in bioinformatics / editoral board, Andreas D Baxevanis [et al] Chapter 13: Unit 13 13. 10.1002/0471250953.bi1303s28 19957274

[36] Gnad F, Young A, Zhou W, Lyle K, Ong CC, et al (2013) Systems-wide analysis of K-Ras, Cdc42, and PAK4 signaling by quantitative phosphoproteomics. Mol Cell Proteomics 12: 2070–2080. 10.1074/mcp.M112.027052 23608596 PMC3734570

[37] Stokes MP, Farnsworth CL, Moritz A, Silva JC, Jia X, et al (2012) PTMScan direct: identification and quantification of peptides from critical signaling proteins by immunoaffinity enrichment coupled with LC-MS/MS. Mol Cell Proteomics 11: 187–201. 10.1074/mcp.M111.015883 22322096 PMC3418847

[38] UniProt C (2014) Activities at the Universal Protein Resource (UniProt). Nucleic Acids Res 42: D191–198. 10.1093/nar/gkt1140 24253303 PMC3965022

[39] Bendtsen JD, Kiemer L, Fausboll A, Brunak S (2005) Non-classical protein secretion in bacteria. BMC Microbiol 5: 58 16212653 10.1186/1471-2180-5-58 PMC1266369

[40] Crooks GE, Hon G, Chandonia JM, Brenner SE (2004) WebLogo: a sequence logo generator. Genome research 14: 1188–1190. 15173120 10.1101/gr.849004 PMC419797

[41] Bhatti MM, Sullivan WJ Jr (2005) Histone acetylase GCN5 enters the nucleus via importin-alpha in protozoan parasite Toxoplasma gondii. J Biol Chem 280: 5902–5908. 15591057 10.1074/jbc.M410656200

[42] Sofroniew MV, Vinters HV (2010) Astrocytes: biology and pathology. Acta Neuropathol 119: 7–35. 10.1007/s00401-009-0619-8 20012068 PMC2799634

[43] Molofsky AV, Krencik R, Ullian EM, Tsai HH, Deneen B, et al (2012) Astrocytes and disease: a neurodevelopmental perspective. Genes Dev 26: 891–907. 10.1101/gad.188326.112 22549954 PMC3347787

[44] Yang JW, Rodrigo R, Felipo V, Lubec G (2005) Proteome analysis of primary neurons and astrocytes from rat cerebellum. J Proteome Res 4: 768–788. 15952724 10.1021/pr049774v

[45] Yang JW, Suder P, Silberring J, Lubec G (2005) Proteome analysis of mouse primary astrocytes. Neurochem Int 47: 159–172. 15908045 10.1016/j.neuint.2005.04.017

[46] Skorupa A, Urbach S, Vigy O, King MA, Chaumont-Dubel S, et al (2013) Angiogenin induces modifications in the astrocyte secretome: relevance to amyotrophic lateral sclerosis. J Proteomics 91: 274–285. 10.1016/j.jprot.2013.07.028 23920243

[47] Yin P, Knolhoff AM, Rosenberg HJ, Millet LJ, Gillette MU, et al (2012) Peptidomic analyses of mouse astrocytic cell lines and rat primary cultured astrocytes. J Proteome Res 11: 3965–3973. 10.1021/pr201066t 22742998 PMC3434970

[48] Dowell JA, Johnson JA, Li L (2009) Identification of astrocyte secreted proteins with a combination of shotgun proteomics and bioinformatics. J Proteome Res 8: 4135–4143. 10.1021/pr900248y 19469553 PMC2866504

[49] Greco TM, Seeholzer SH, Mak A, Spruce L, Ischiropoulos H (2010) Quantitative mass spectrometry-based proteomics reveals the dynamic range of primary mouse astrocyte protein secretion. J Proteome Res 9: 2764–2774. 10.1021/pr100134n 20329800 PMC2866110

[50] Han D, Jin J, Woo J, Min H, Kim Y (2014) Proteomic analysis of mouse astrocytes and their secretome by a combination of FASP and StageTip-based, high pH, reversed-phase fractionation. Proteomics 14: 1604–1609. 10.1002/pmic.201300495 24753479

[51] Kazantsev AG, Thompson LM (2008) Therapeutic application of histone deacetylase inhibitors for central nervous system disorders. Nat Rev Drug Discov 7: 854–868. 10.1038/nrd2681 18827828

[52] Harrison IF, Dexter DT (2013) Epigenetic targeting of histone deacetylase: therapeutic potential in Parkinson's disease? Pharmacol Ther 140: 34–52. 10.1016/j.pharmthera.2013.05.010 23711791

[53] Burli RW, Luckhurst CA, Aziz O, Matthews KL, Yates D, et al (2013) Design, synthesis, and biological evaluation of potent and selective class IIa histone deacetylase (HDAC) inhibitors as a potential therapy for Huntington's disease. J Med Chem 56: 9934–9954. 10.1021/jm4011884 24261862

[54] Palfreyman JW, Thomas DG, Ratcliffe JG, Graham DI (1979) Glial fibrillary acidic protein (GFAP): purification from human fibrillary astrocytoma, development and validation of a radioimmunoassay for GFAP-like immunoactivity. J Neurol Sci 41: 101–113. 438840 10.1016/0022-510x(79)90144-8

[55] Eng LF, Vanderhaeghen JJ, Bignami A, Gerstl B (1971) An acidic protein isolated from fibrous astrocytes. Brain Res 28: 351–354. 5113526 10.1016/0006-8993(71)90668-8

[56] Lee KK, Workman JL (2007) Histone acetyltransferase complexes: one size doesn't fit all. Nat Rev Mol Cell Biol 8: 284–295. 17380162 10.1038/nrm2145

[57] Shahbazian MD, Grunstein M (2007) Functions of site-specific histone acetylation and deacetylation. Annu Rev Biochem 76: 75–100. 17362198 10.1146/annurev.biochem.76.052705.162114

[58] Kim SC, Sprung R, Chen Y, Xu Y, Ball H, et al (2006) Substrate and functional diversity of lysine acetylation revealed by a proteomics survey. Mol Cell 23: 607–618. 16916647 10.1016/j.molcel.2006.06.026

[59] Foster DB, Liu T, Rucker J, O'Meally RN, Devine LR, et al (2013) The cardiac acetyl-lysine proteome. PLoS One 8: e67513 10.1371/journal.pone.0067513 23844019 PMC3699649

[60] Andoh A, Fujiyama Y, Shimada M, Bamba T (2000) Modulation of complement component (C3 and factor B) biosynthesis by a histone deacetylase inhibitor in human intestinal epithelial cells. Int J Mol Med 6: 51–54. 10851266

[61] Abdelmagid SM, Barbe MF, Rico MC, Salihoglu S, Arango-Hisijara I, et al (2008) Osteoactivin, an anabolic factor that regulates osteoblast differentiation and function. Exp Cell Res 314: 2334–2351. 10.1016/j.yexcr.2008.02.006 18555216

[62] Nakano Y, Suzuki Y, Takagi T, Kitashoji A, Ono Y, et al (2014) Glycoprotein nonmetastatic melanoma protein B (GPNMB) as a novel neuroprotective factor in cerebral ischemia-reperfusion injury. Neuroscience 277: 123–131. 10.1016/j.neuroscience.2014.06.065 25010402

[63] Hornbeck PV, Chabra I, Kornhauser JM, Skrzypek E, Zhang B (2004) PhosphoSite: A bioinformatics resource dedicated to physiological protein phosphorylation. Proteomics 4: 1551–1561. 15174125 10.1002/pmic.200300772

[64] Piperno G, LeDizet M, Chang XJ (1987) Microtubules containing acetylated alpha-tubulin in mammalian cells in culture. J Cell Biol 104: 289–302. 2879846 10.1083/jcb.104.2.289 PMC2114420

[65] Janke C (2014) The tubulin code: molecular components, readout mechanisms, and functions. J Cell Biol 206: 461–472. 10.1083/jcb.201406055 25135932 PMC4137062

[66] Huang da W, Sherman BT, Lempicki RA (2009) Systematic and integrative analysis of large gene lists using DAVID bioinformatics resources. Nat Protoc 4: 44–57. 10.1038/nprot.2008.211 19131956

[67] Huang da W, Sherman BT, Lempicki RA (2009) Bioinformatics enrichment tools: paths toward the comprehensive functional analysis of large gene lists. Nucleic Acids Res 37: 1–13. 10.1093/nar/gkn923 19033363 PMC2615629

[68] Cahoy JD, Emery B, Kaushal A, Foo LC, Zamanian JL, et al (2008) A transcriptome database for astrocytes, neurons, and oligodendrocytes: a new resource for understanding brain development and function. J Neurosci 28: 264–278. 10.1523/JNEUROSCI.4178-07.2008 18171944 PMC6671143

[69] Colaert N, Helsens K, Martens L, Vandekerckhove J, Gevaert K (2009) Improved visualization of protein consensus sequences by iceLogo. Nature methods 6: 786–787. 10.1038/nmeth1109-786 19876014

[70] Lundby A, Lage K, Weinert BT, Bekker-Jensen DB, Secher A, et al (2012) Proteomic analysis of lysine acetylation sites in rat tissues reveals organ specificity and subcellular patterns. Cell Rep 2: 419–431. 10.1016/j.celrep.2012.07.006 22902405 PMC4103158

[71] Hu X, Zhang P, Xu Z, Chen H, Xie X (2013) GPNMB enhances bone regeneration by promoting angiogenesis and osteogenesis: potential role for tissue engineering bone. J Cell Biochem 114: 2729–2737. 10.1002/jcb.24621 23794283

[72] Chung JS, Dougherty I, Cruz PD Jr, Ariizumi K (2007) Syndecan-4 mediates the coinhibitory function of DC-HIL on T cell activation. J Immunol 179: 5778–5784. 17947650 10.4049/jimmunol.179.9.5778

[73] Maric G, Rose AA, Annis MG, Siegel PM (2013) Glycoprotein non-metastatic b (GPNMB): A metastatic mediator and emerging therapeutic target in cancer. Onco Targets Ther 6: 839–852. 10.2147/OTT.S44906 23874106 PMC3711880

[74] Tanaka H, Shimazawa M, Kimura M, Takata M, Tsuruma K, et al (2012) The potential of GPNMB as novel neuroprotective factor in amyotrophic lateral sclerosis. Sci Rep 2: 573 10.1038/srep00573 22891158 PMC3417778

[75] Li B, Castano AP, Hudson TE, Nowlin BT, Lin SL, et al (2010) The melanoma-associated transmembrane glycoprotein Gpnmb controls trafficking of cellular debris for degradation and is essential for tissue repair. FASEB J 24: 4767–4781. 10.1096/fj.10-154757 20709912 PMC2992370

[76] Yang C, Wu J, Zheng YG (2012) Function of the active site lysine autoacetylation in Tip60 catalysis. PLoS One 7: e32886 10.1371/journal.pone.0032886 22470428 PMC3314657

[77] Patel JH, Du Y, Ard PG, Phillips C, Carella B, et al (2004) The c-MYC oncoprotein is a substrate of the acetyltransferases hGCN5/PCAF and TIP60. Mol Cell Biol 24: 10826–10834. 15572685 10.1128/MCB.24.24.10826-10834.2004 PMC533976

[78] Lang C, Hildebrandt A, Brand F, Opitz L, Dihazi H, et al (2012) Impaired chromatin remodelling at STAT1-regulated promoters leads to global unresponsiveness of Toxoplasma gondii-infected macrophages to IFN-gamma. PLoS Pathog 8: e1002483 10.1371/journal.ppat.1002483 22275866 PMC3262016

[79] Whitesell L, Lindquist SL (2005) HSP90 and the chaperoning of cancer. Nat Rev Cancer 5: 761–772. 16175177 10.1038/nrc1716

[80] Mollapour M, Neckers L (2012) Post-translational modifications of Hsp90 and their contributions to chaperone regulation. Biochim Biophys Acta 1823: 648–655. 10.1016/j.bbamcr.2011.07.018 21856339 PMC3226900

[81] Wagner SA, Beli P, Weinert BT, Scholz C, Kelstrup CD, et al (2012) Proteomic analyses reveal divergent ubiquitylation site patterns in murine tissues. Mol Cell Proteomics 11: 1578–1585. 10.1074/mcp.M112.017905 22790023 PMC3518112

[82] Kim W, Bennett EJ, Huttlin EL, Guo A, Li J, et al (2011) Systematic and quantitative assessment of the ubiquitin-modified proteome. Mol Cell 44: 325–340. 10.1016/j.molcel.2011.08.025 21906983 PMC3200427

[83] Vogel R, Wiesinger H, Hamprecht B, Dringen R (1999) The regeneration of reduced glutathione in rat forebrain mitochondria identifies metabolic pathways providing the NADPH required. Neurosci Lett 275: 97–100. 10568508 10.1016/s0304-3940(99)00748-x

[84] Deponte M (2013) Glutathione catalysis and the reaction mechanisms of glutathione-dependent enzymes. Biochim Biophys Acta 1830: 3217–3266. 10.1016/j.bbagen.2012.09.018 23036594

[85] Yu W, Dittenhafer-Reed KE, Denu JM (2012) SIRT3 protein deacetylates isocitrate dehydrogenase 2 (IDH2) and regulates mitochondrial redox status. J Biol Chem 287: 14078–14086. 10.1074/jbc.M112.355206 22416140 PMC3340192

[86] Wurm T, Wright DG, Polakowski N, Mesnard JM, Lemasson I (2012) The HTLV-1-encoded protein HBZ directly inhibits the acetyl transferase activity of p300/CBP. Nucleic Acids Res. 10.1093/nar/gks244 PMC3401433 22434882

[87] Ablack JN, Cohen M, Thillainadesan G, Fonseca GJ, Pelka P, et al (2012) Cellular GCN5 Is a Novel Regulator of Human Adenovirus E1A-Conserved Region 3 Transactivation. J Virol 86: 8198–8209. 10.1128/JVI.00289-12 22623781 PMC3421684

[88] Terreni M, Valentini P, Liverani V, Gutierrez MI, Di Primio C, et al (2010) GCN5-dependent acetylation of HIV-1 integrase enhances viral integration. Retrovirology 7: 18 10.1186/1742-4690-7-18 20226045 PMC2848186

[89] Col E, Caron C, Chable-Bessia C, Legube G, Gazzeri S, et al (2005) HIV-1 Tat targets Tip60 to impair the apoptotic cell response to genotoxic stresses. EMBO J 24: 2634–2645. 16001085 10.1038/sj.emboj.7600734 PMC1176461

[90] Lodewick J, Lamsoul I, Polania A, Lebrun S, Burny A, et al (2009) Acetylation of the human T-cell leukemia virus type 1 Tax oncoprotein by p300 promotes activation of the NF-kappaB pathway. Virology 386: 68–78. 10.1016/j.virol.2008.12.043 19200568 PMC2834172

[91] Cereseto A, Manganaro L, Gutierrez MI, Terreni M, Fittipaldi A, et al. (2005) Acetylation of HIV-1 integrase by p300 regulates viral integration. Embo J. 10.1038/sj.emboj.7600770 PMC1201351 16096645

[92] Chi H, Flavell RA (2008) Acetylation of MKP-1 and the control of inflammation. Science signaling 1: pe44 10.1126/scisignal.141pe44 18922786 PMC2613485

[93] Munoz-Fontela C, Gonzalez D, Marcos-Villar L, Campagna M, Gallego P, et al (2011) Acetylation is indispensable for p53 antiviral activity. Cell cycle 10: 3701–3705. 10.4161/cc.10.21.17899 22033337 PMC3685621

[94] Wang X, Li D, Qu D, Zhou D (2010) Involvement of TIP60 acetyltransferase in intracellular Salmonella replication. BMC Microbiol 10: 228 10.1186/1471-2180-10-228 20796290 PMC3313078

[95] Wu S, Ye Z, Liu X, Zhao Y, Xia Y, et al (2010) Salmonella typhimurium infection increases p53 acetylation in intestinal epithelial cells. Am J Physiol Gastrointest Liver Physiol 298: G784–794. 10.1152/ajpgi.00526.2009 20224008 PMC2867426

[96] Norris KL, Lee JY, Yao TP (2009) Acetylation goes global: the emergence of acetylation biology. Science signaling 2: pe76 10.1126/scisignal.297pe76 19920250 PMC2812806

[97] Du J, An R, Chen L, Shen Y, Chen Y, et al (2014) Toxoplasma gondii virulence factor ROP18 inhibits the host NF-kappaB pathway by promoting p65 degradation. J Biol Chem 289: 12578–12592. 10.1074/jbc.M113.544718 24648522 PMC4007449

[98] Alaganan A, Fentress SJ, Tang K, Wang Q, Sibley LD (2014) Toxoplasma GRA7 effector increases turnover of immunity-related GTPases and contributes to acute virulence in the mouse. Proc Natl Acad Sci U S A 111: 1126–1131. 10.1073/pnas.1313501111 24390541 PMC3903209

[99] Braun L, Brenier-Pinchart MP, Yogavel M, Curt-Varesano A, Curt-Bertini RL, et al (2013) A Toxoplasma dense granule protein, GRA24, modulates the early immune response to infection by promoting a direct and sustained host p38 MAPK activation. J Exp Med 210: 2071–2086. 10.1084/jem.20130103 24043761 PMC3782045

